# PhyTB: Phylogenetic tree visualisation and sample positioning for *M. tuberculosis*

**DOI:** 10.1186/s12859-015-0603-3

**Published:** 2015-05-13

**Authors:** Ernest D Benavente, Francesc Coll, Nick Furnham, Ruth McNerney, Judith R Glynn, Susana Campino, Arnab Pain, Fady R Mohareb, Taane G Clark

**Affiliations:** Faculty of Infectious and Tropical Diseases, London School of Hygiene & Tropical Medicine, Keppel St, London, UK; Engineering Sciences Division, School of Engineering, Cranfield University, Cranfield, UK; Faculty of Epidemiology and Population Health, London School of Hygiene & Tropical Medicine, Keppel St, London, UK; Wellcome Trust Sanger Institute, Hinxton, Cambridge UK; Biological and Environmental Sciences and Engineering Division, King Abdullah University of Science and Technology, Thuwal, Kingdom of Saudi Arabia

**Keywords:** Tuberculosis, Phylogeny, Sequencing

## Abstract

**Background:**

Phylogenetic-based classification of *M. tuberculosis* and other bacterial genomes is a core analysis for studying evolutionary hypotheses, disease outbreaks and transmission events. Whole genome sequencing is providing new insights into the genomic variation underlying intra- and inter-strain diversity, thereby assisting with the classification and molecular barcoding of the bacteria. One roadblock to strain investigation is the lack of user-interactive solutions to interrogate and visualise variation within a phylogenetic tree setting.

**Results:**

We have developed a web-based tool called *PhyTB* (http://pathogenseq.lshtm.ac.uk/phytblive/index.php) to assist phylogenetic tree visualisation and identification of *M. tuberculosis* clade-informative polymorphism. Variant Call Format files can be uploaded to determine a sample position within the tree. A map view summarises the geographical distribution of alleles and strain-types. The utility of the *PhyTB* is demonstrated on sequence data from 1,601 *M. tuberculosis* isolates.

**Conclusion:**

*PhyTB* contextualises *M. tuberculosis* genomic variation within epidemiological, geographical and phylogenic settings. Further tool utility is possible by incorporating large variants and phenotypic data (e.g. drug-resistance profiles), and an assessment of genotype-phenotype associations. Source code is available to develop similar websites for other organisms (http://sourceforge.net/projects/phylotrack).

## Background

Strain-specific genomic diversity in the *Mycobacterium tuberculosis* complex (MTBC) is an important factor in tuberculosis pathogenesis that may affect virulence, transmissibility, host response and emergence of drug resistance [1,2]. Some modern strains (e.g. Beijing, Euro-American, Haarlem) are believed to exhibit more virulent phenotypes compared to ancient ones (e.g. East African, Indian, *M. africanum*) [2]. *M. tuberculosis* is relatively clonal, with little recombination and a low mutation rate [3]. Like other bacterial genomic settings, the construction of phylogenetic trees using sequence data facilitates taxonomic localisation and the evolutionary analysis. The growing availability of *M. tuberculosis* whole genome sequences is leading to the full characterisation of single nucleotide polymorphisms (SNPs) and other nucleotide variation, such as insertions and deletions (indels). A SNP–based barcode has been developed to discriminate strain-types [2]. Trees constructed using genome-wide variation have greater discriminatory power than traditional genotyping approaches such as MIRU-VNTR and spoligotyping [4]. Clades reflecting strain type variations may be used to investigate disease outbreaks or transmission events, where samples are identified through apparent identical genomic signatures [5,6]. The tree also provides a structure to identify variants that can be used to investigate clinically important traits such as drug resistance [5]. The primary mechanism for acquiring resistance is the accumulation of point mutations in genes coding for drug-targets or -converting enzymes (e.g. *katG, inhA, rpoB, pncA, embB, rrs, gyrA, gyrB* genes) [7], and these mutations may exist in multiple lineages in the tree, reflecting homoplasy events. Some mutations thought to be related to drug resistance are actually not, but instead strain-informative [2]. With the increased application of sequencing technologies within clinical and microbiological research settings, it is important that informatic tools are available to identify informative strain-type and drug resistance related variants. Web-browsers for the visualisation of *M. tuberculosis* genomic variation exist [8-10], but there is limited connectivity with phylogenetic trees and downstream analysis, especially involving strain-types and drug resistance. In addition, there is little provision for uploading new data, such as standard variant call files (VCFs) (www.htslib.org). Here we present the *PhyTB* tool, which facilitates the phylogenetic exploration of *M. tuberculosis* isolates, including the display of clade-specific informative and drug resistance markers and their genomic annotation. Using the browser, it is possible to upload multiple standard genomic variant call files (VCF format) to identify the closest relative within the *M. tuberculosis* complex global phylogeny, thereby potentially assisting their interpretation in a clinical or epidemiological context. Source code is available to facilitate the development of sites for other organisms with genomes that can be represented in a phylogeny.

## Implementation

*PhyTB* is a JavaScript–based web-browsing tool that uses the *D3.js* library for data visualization [11] and the *JBrowse* tool for genome browser representation [12]. The source code has been integrated and called *PhyloTrack*, enabling websites for other organisms to be developed (http://sourceforge.net/projects/phylotrack). The software requires a phylogenetic tree of the common Newick data format as input, and tab delimited meta data files for samples, clade-defining nodes and clade colour definitions. The phylogenetic tree was constructed using 91 k SNPs mapped against the H37Rv reference genome [Genbank:NC_000962.3]. These SNPs were identified using a combination of *bwa-mem* alignment software (bio-bwa.sourceforge.net) and the *SAMtools/BCFtools* suite (samtools.sourceforge.net) complemented by *GATK* (https://www.broadinstitute.org/gatk/). Variants at Q-score of 30 or more were then selected from the intersection dataset between those obtained from both *SAMtools* and *GATK*. SNPs in non-unique regions, including repeat regions in PE/PPE genes were removed (see [2] for details). The best-scoring maximum likelihood phylogenetic tree was computed using RAxML v7.4.2 (http://sco.h-its.org/exelixis/web/software/raxml/index.html) based on 91,648 sites spanning the whole genome. Given the considerable size of the dataset (1,601 samples, 91,648 SNP sites), the rapid bootstrapping algorithm (N = 100, ×= 12,345) combined with maximum likelihood search was chosen to construct the phylogenetic tree including only branches with bootstrap values greater than 95%. The resulting tree was rooted on *M. canettii* [Genbank: NC_019950.1] and nodes were annotated. Subsequently, the ancestral sequence at all internal nodes was computed using DnaPars from the Phylip package (http://evolution.genetics.washington.edu/phylip/). The main lineage- and sublineage-defining nodes were initially identified from the tree, based on the spoligotypes in each clade. Informative markers at each node in the phylogenetic tree are stored in VCF files and displayed, highlighting clade-defining polymorphism. This functionality has been implemented using the *tabix* tool [13] on the server side. The informative variants have been established by comparing allele frequencies between strain-types using ancestral node comparisons [2]. Perl scripts used to generate these data is included within the *PhyloTrack* package. These include scripts to convert a tree in JSON format for use by the *D3.js* library, produce metadata for each node, and process VCF files containing information for each node and SNP. VCF files containing clade informative and drug resistance markers [2] are compressed using *bgzip* and indexed using *tabix* to improve computational efficiency, as well as to act as a database. Variants in user uploaded VCF files are compared to those in the database to establish a sample’s position within the tree. Using node-specific SNPs, the possible paths inside the tree are reconstructed, and the one with the most SNP matches is reported. *PhyTB*’s map view shows allele and strain-type frequencies by geographical location, developed from *PolyTB* source code [9].

## Results and discussion

**Figure 1:**
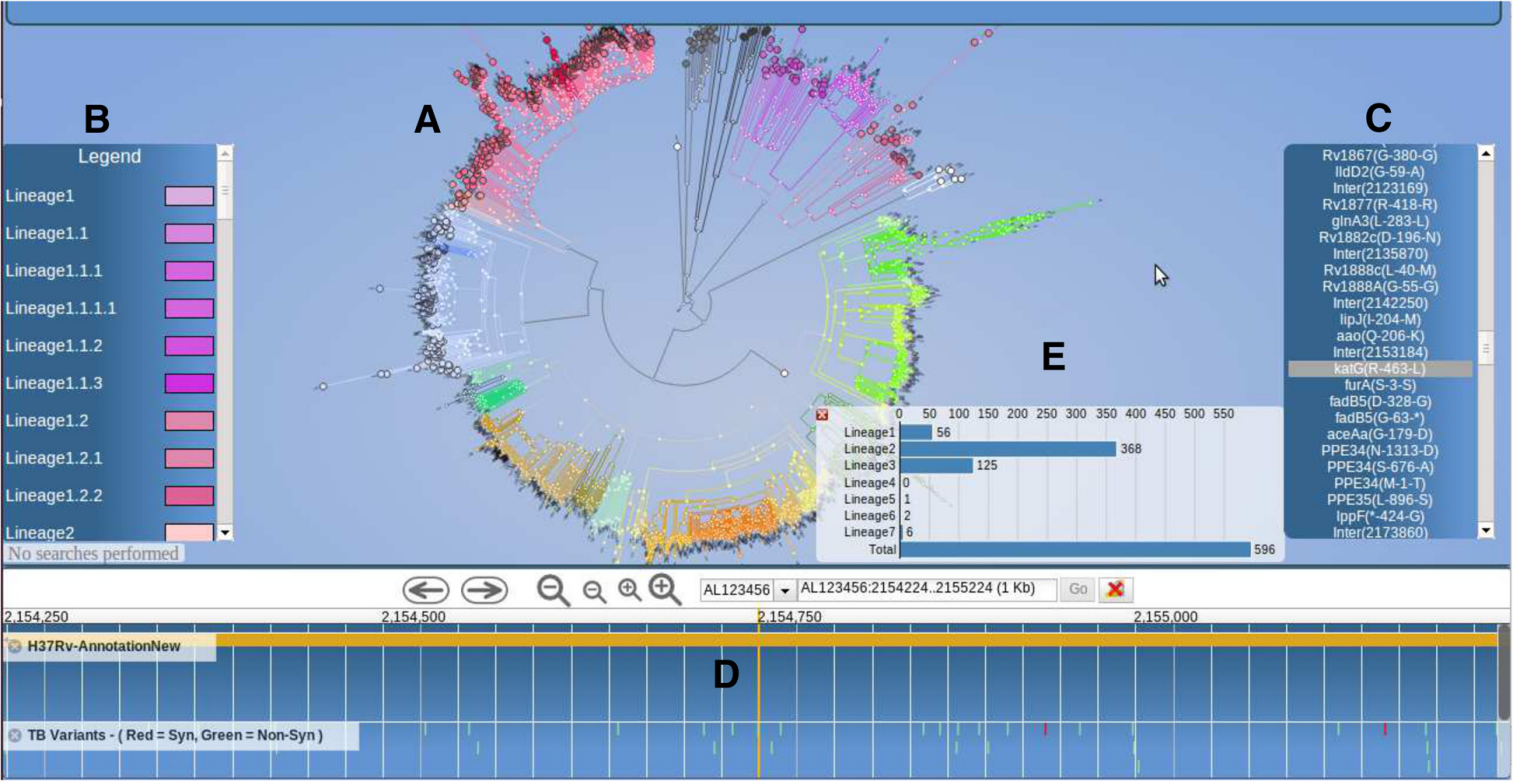
PhyTB screenshot: A phylogenetic tree for the 1,601 *M. tuberculosis* isolates **(A)**, with each lineage colour coded **(B)**. A selected SNP R463L in the *katG* gene (associated with isoniazid resistance) **(C)**, is located at position 2,152,224, **(D)** and present across all but one lineage (4) **(E)**.

**Figure 2:**
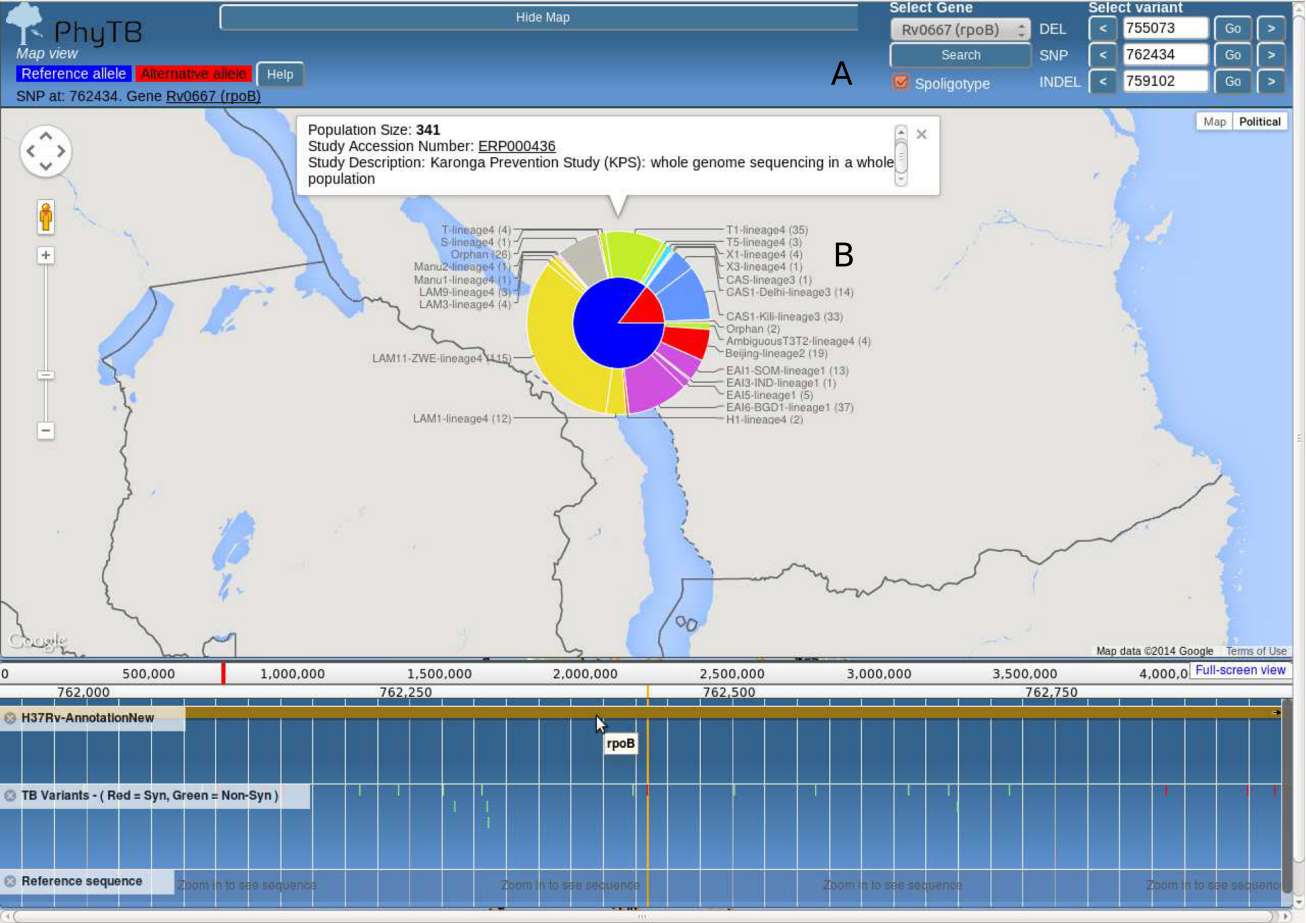
PhyTB screenshot: A map view showing the frequency of the G876G SNP in the *rpoB* gene (searchable from **(A)**) and its association with lineage 3 strain-types in Malawi. Pie chart **(B)** shows the non-reference allele frequency (red segment, inner circle) is linked to the CAS spoligotype (blue segment, outer circle).

**Figure 3:**
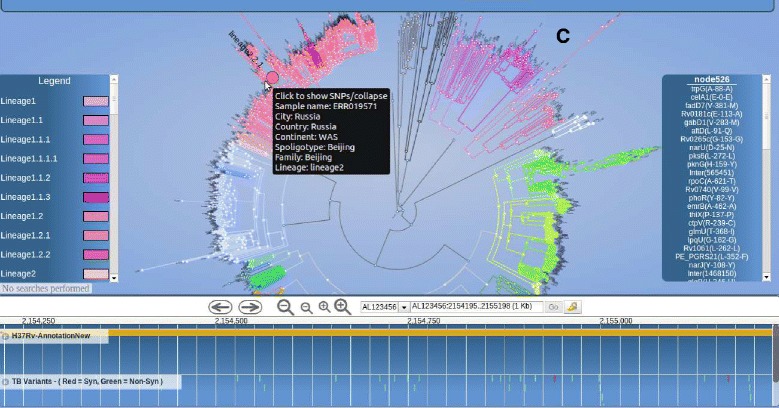
PhyTB screenshot: A Russian sample (ERR019571) is located in a Beijing clade in lineage 2.2.1 **(C)**, established using variants uploaded in a Variant Call Format file.

*PhyTB* uses 1,601 global MTBC whole-genome sequences from 11 studies with representation across all 7 major lineages (lineage 1 - 7.6%, 2 - 24.3%, 3 - 11.8%, 4 - 53.5%, 5-7 2.8%). The phylogenetic tree constructed using the 91 k SNPs shows the expected clustering by lineage and strain-type (Figure 1). SNP information is displayed at internal nodes of the tree, therefore distinguishing between unique strain-defining mutations from those arising in multiple branches (homoplastic mutations). The homoplastic mutations arise due to recombination or convergent evolution, potentially related to drug resistance. Figure 1 shows a deep phylogenetic SNP (R463L) in the *katG* gene that is present across all lineages except lineage 4. This SNP has been historically and mistakenly thought to cause isoniazid resistance. *PhyTB* displays whether polymorphisms have been previously related to drug resistance [14] or are strain informative [2] in tracks, and meta data (e.g. codon, amino acid) is shown by selecting the polymorphism of interest. It is possible to move from the tree view to a geographical map showing allele frequencies. A map view, accessed through the genome browser located below the tree, shows a SNP at position 762,434 in *rpoB*, a gene associated with rifampicin resistance. The alternative allele leads to a synonymous mutation (G876G) that is fixed in CAS (lineage 3) strains in Malawi (Figure 2) and all other study sites. To demonstrate the VCF positioning functionality, we used 100 *M. tuberculosis* samples [ENA:ERP000192] of known strain-type [9], not included in the phylogeny. It was possible to unambiguously position all of them in the tree. Figure 3 shows the result of uploading the VCF file for a Russian sample [ENA:ERR019571], which has 5067 SNPs, allowing it to be positioned correctly in a Beijing clade.

## Conclusion

The *PhyTB* web-browser attempts to contextualise TB genomic variation within epidemiological, geographical and phylogenic settings. To assist with integrating such data for other organisms, we provide the source code, which has been packaged in the PhyloTrack library. In pathogenic bacteria like *M. tuberculosis*, data integration is crucial to distinguish drug-resistance mutations from phylogenetic markers, to study the transmission of outbreak strains, to detect the source of an infection, inform patient management and design appropriate infection control measures (e.g. rapid tests). Further tool utility is possible by extending it to incorporate large variants and phenotypic data (e.g. drug-resistance profiles).

## Availability and requirements

**Project name:***PhyTB***Project home page:**http://pathogenseq.lshtm.ac.uk/phytblive/index.php**Source code:***PhyloTrack* - http://sourceforge.net/projects/phylotrack**Operating system(s)**: Platform independent **Programming language**: JavaScript and Perl **Other requirements:** None **License:** None **Any restrictions to use by non-academics:** None
